# Maternal High Fat Diet Is Associated with Decreased Plasma n–3 Fatty Acids and Fetal Hepatic Apoptosis in Nonhuman Primates

**DOI:** 10.1371/journal.pone.0017261

**Published:** 2011-02-25

**Authors:** Wilmon F. Grant, Melanie B. Gillingham, Ayesha K. Batra, Natasha M. Fewkes, Sarah M. Comstock, Diana Takahashi, Theodore P. Braun, Kevin L. Grove, Jacob E. Friedman, Daniel L. Marks

**Affiliations:** 1 Neuroscience Graduate Program, Oregon Health & Science University, Portland, Oregon, United States of America; 2 Department of Pediatrics, Oregon Health & Science University, Portland, Oregon, United States of America; 3 Human Investigations Program of the Oregon Clinical and Translational Research Institute, Oregon Health & Science University, Portland, Oregon, United States of America; 4 Department of Molecular and Medical Genetics, Oregon Health & Science University, Portland, Oregon, United States of America; 5 Center for the Study of Weight Regulation, Oregon Health & Science University, Portland, Oregon, United States of America; 6 Oregon National Primate Research Center, Oregon Health & Science University, Portland, Oregon, United States of America; 7 Department of Pediatrics, University of Colorado Denver, Aurora, Colorado, United States of America; Indiana University, United States of America

## Abstract

To begin to understand the contributions of maternal obesity and over-nutrition to human development and the early origins of obesity, we utilized a non-human primate model to investigate the effects of maternal high-fat feeding and obesity on breast milk, maternal and fetal plasma fatty acid composition and fetal hepatic development. While the high-fat diet (HFD) contained equivalent levels of n-3 fatty acids (FA's) and higher levels of n-6 FA's than the control diet (CTR), we found significant decreases in docosahexaenoic acid (DHA) and total n-3 FA's in HFD maternal and fetal plasma. Furthermore, the HFD fetal plasma n-6∶n-3 ratio was elevated and was significantly correlated to the maternal plasma n-6∶n-3 ratio and maternal hyperinsulinemia. Hepatic apoptosis was also increased in the HFD fetal liver. Switching HFD females to a CTR diet during a subsequent pregnancy normalized fetal DHA, n-3 FA's and fetal hepatic apoptosis to CTR levels. Breast milk from HFD dams contained lower levels of eicosopentanoic acid (EPA) and DHA and lower levels of total protein than CTR breast milk. This study links chronic maternal consumption of a HFD with fetal hepatic apoptosis and suggests that a potentially pathological maternal fatty acid milieu is replicated in the developing fetal circulation in the nonhuman primate.

## Introduction

Over the last twenty years, obesity has dramatically increased in the United States across every ethnic group studied [1], [2]. Women of childbearing age have not been spared from this upsurge, as nearly 50% of all women of childbearing age are either overweight or obese and one-third have body mass indexes (BMI) of 30 or higher [3], [4]. A particularly concerning part of the emerging epidemic of obesity is the increasing rise in the percentage of children and adolescents that are either overweight or at risk for overweight [1], [2], [5]. In addition, diseases once only found in adults are occurring with greater frequency in pediatric populations. Type 2 diabetes mellitus as well as secondary co-morbidities such as hypertension, non-alcoholic fatty liver disease, hyperlipidemia, and metabolic syndrome are now becoming increasingly common in children [6], [7].

The increasing prevalence of metabolic diseases and obesity in children is most often attributed to a combination of an energy conserving, or ‘thrifty’ genotype, with a prevalent imbalance of nutrient intake and expenditure in the developed world. An emerging body of evidence also suggests that our ability to respond to metabolic challenges during postnatal life is modified by environmental influences during fetal development. Fetal development is a critical period when exposure to environmental insults *in-utero* has lifelong effects on the structure and function of organs, tissues and body systems in the offspring.

There is strong evidence in humans that maternal nutrient deprivation during pregnancy can program adipocyte metabolism and fat mass towards a propensity for obesity, and lead to a wide range of developmental effects in the offspring [8]–[15]. In addition, maternal obesity and gestational diabetes mellitus (GDM) during pregnancy has also been implicated in the development of metabolic disorders in offspring, including macrosomia, impaired glucose tolerance, and a higher risk of developing obesity and diabetes as adults [16]–[25]. While epidemiological evidence has shown that the intrauterine environment has profound effects on fetal growth and the programming of childhood weight, the mechanisms underlying metabolic programming are poorly understood, particularly in humans.

Rodent studies have demonstrated that a maternal high fat diet during gestation and lactation, or overfeeding during the postnatal period, alters the development of the pancreas and liver as well as central and peripheral nervous systems involved in energy homeostasis [26]–[40]. While extremely valuable, these rodent studies do not address the fact that there are critical developmental differences between rodents and primates, including both humans and nonhuman primates (NHP). For example, the development of important central circuits regulating appetite and metabolism occurs prenatally in humans and NHP, while rodent maturation of these systems primarily occurs postnatally [41]–[45]. Furthermore, the macro- and micro-architecture of the placenta is markedly different between rodents and humans, and this has important implications for fetal nutrient transfer during development (reviewed in [46]). Therefore, studies designed to provide mechanistic links between the maternal gestational metabolic environment and fetal metabolic programming, in support of previous human epidemiological observations, requires animal models that closely resemble human development.

To this end, we have utilized a unique non-human primate model of maternal high fat/calorie diet-induced obesity (in the absence of gestational diabetes) to address the impact that *chronic* maternal consumption of a high-fat diet (HFD) may have on metabolic programming [47]. We acknowledge that ‘high-fat’ is a phrase that simplifies the complex nature of our dietary intervention and therefore have supplied a detailed analysis of the dietary constituents. Nonetheless, this model utilizes chronic high fat feeding, and the level and composition of dietary fat are not outside the norms for a modern western diet [48].

Our group has previously shown that a maternal HFD alters fetal development and expression of hypothalamic neuropeptides in the context of hypothalamic inflammation, as well as inducing changes in the central serotonergic system [49], [50]. In the liver, HFD fetuses had premature gluconeogenic gene expression, steatosis, elevated triglyceride content, and oxidative stress. In addition, epigenetic changes and altered circadian gene expression has been shown in the HFD fetal liver [51], [52]. HFD fetal plasma contained elevated levels of inflammatory cytokines, and elevated triglycerides and glycerol that were highly correlated to maternal levels. Other work with this model has shown that alterations in serum metabolite profiles are present in HFD fetuses [53]. The majority of these changes were observed irrespective of maternal obesity or maternal insulin resistance status and persisted into the early postnatal period. Importantly, switching HFD mothers to a control diet during pregnancy alone (diet-reversal; REV) reversed a number of the observed fetal hepatic pathologies towards control levels [47], [52].

In the present study, we further characterize our NHP model of maternal HFD induced obesity [47], by a detailed analysis of fatty acids in the diet, maternal plasma, breastmilk, and fetal plasma. In addition, we extend previous findings by evaluating the effects that a maternal HFD has on inflammation and apoptosis in the fetal liver. Our results show that the experimental maternal HFD leads to increased apoptosis in the developing fetal liver. In addition, maternal and fetal HFD plasma have reduced levels of circulating n-3 fatty acids when compared to CTR. Importantly, we demonstrate that a maternal dietary intervention during pregnancy (REV) normalized fetal hepatic apoptosis and returned plasma n-3 fatty acids to CTR levels in dams and fetuses. These data support the idea that the placenta does not protect the developing fetus from a pro-inflammatory maternal lipid milieu. Because these effects are associated with maternal diet during gestation and lactation, and some are reversed with dietary manipulation limited to these intervals, these data have critical public health implications.

## Materials and Methods

### Macaque model of maternal overnutrition

All animal procedures have undergone an extensive review process and were in accordance with the guidelines of Institutional Animal Care and Use Committee of the Oregon National Primate Research Center (ONPRC) and Oregon Health & Science University. Protocols involved in this study were developed to ameliorate suffering and have been approved under IACUC ID number: IS00000224 (0622 for internal purposes). The Animal Care and Use Program at the ONPRC abides by the Animal Welfare Act and Regulations (CFR 9, Ch 1, Subchapter A) enforced by the USDA, the Public Health Service Policy on Humane Care and Use of Laboratory Animals, in accordance with the *Guide for the Care and Use of Laboratory Animals* of the National Institutes of Health, and the recommendations of the Weatherall report; *The Use of Non-human Primates in Research*.

Japanese macaques matched for age (5–7 years at start) and weight (7–9 Kg) were randomly assigned to two dietary groups in the fall of 2002: 1: Control diet (CTR; 13% of calories from fat; Monkey Diet no. 5052, Lab Diet, Richmond, IN, USA) or 2: High-fat diet (HFD; 35.2% of calories from fat; Custom Diet 5A1F, Test Diet, Richmond, IN, USA). The HFD also included calorically dense treats made with peanut butter. Both diets are sufficient in vitamin, mineral, and protein content for normal growth. Prior to this study, all animals were maintained on standard monkey chow in large outdoor enclosures and were naive to any experimental protocols.

Manufacturers specifications provided for both diets show that the total metabolizable energy content of the CTR chow was 2.87 kcal/g and was apportioned at 26.8% energy from protein, 58.5% energy from carbohydrate, and 14.7% energy from fat. The main source of fat in the CTR diet was soybean oil. The total energy content of the maternal HFD chow was 4.2 kcal/g and was apportioned at 16.7% energy from protein, 51.5% energy from carbohydrate, and 31.8% energy from fat. The main sources of fat in the HFD were lard, animal fat, butter and safflower oil.

The animals were group housed and had *ad libitum* access to food and water. The group housing is important as it provides for normal social behavior and exercise, which contribute to the psychological well being of the animals and more closely resembles the human condition. However, because the animals are group housed it is not possible to determine individual food/calorie intake. For maternal plasma studies, 11 CTR, 6 HFD, and 7 REV dams were used.

Each maternal group was housed with two males so that pregnancies would occur during the yearly breeding season (November–February). The females were checked each successive year for pregnancies starting in November by ultrasound, which allows an estimate of gestational age ±5 days. Twice a year the animals underwent IV glucose tolerance tests (IVGTT) (**Methods S1a**), once during the late summer (nonpregnant state) and once during the early 3rd trimester of pregnancy. All of the above procedures were done under ketamine sedation (5–10 mg/kg).

For our studies, ONPRC veterinarians terminated singleton pregnancies from dams by cesarean section at gestational day 130 (G130), as determined by ultrasound. Pregnant dams were fasted overnight for approximately 16 hours prior to surgical procedure. Females were initially sedated with ketamine hydrochloride (100 mg/ml) at a dose of 10–15 mg/kg. Once animals were sedated they were delivered to the surgical area and placed on isoflurane gas; induced at 3%, then maintained at 1.0–1.5%. Cesarean sections were performed by trained ONPRC veterinarians and their staff, and occurred on scheduled days between 10:00 and 10:30 am.

Pre and post-operative care was maintained by the ONPRC veterinary staff. Immediately prior to the cesarean section animals received an intravenous dose of hydromorphone (0.5 mg if under 10 kg, 1.0 mg if over 10 kg). An additional intravenous dose of hydromorphone was administered post-operatively, usually within an hour after the start of the procedure. For the remainder of the day following the cesarean section, intravenous hydromorphone was given at 4:00 pm and again at 8:00 pm in combination with buprenorphine (0.3 mg IM). The following day, hydromorphone was administered at 8:00 am, 12:00 pm, 4:00 pm and then again with buprenorphine at 8:00 pm. Animals remained in the surgical ICU area for approximately 7 days under close veterinary observation and were then released back into their group.

After cesarean section, fetuses were deeply anesthetized with sodium pentobarbital (>30 mg/kg i.v.) and exsanguinated. All peripheral tissues and brain were removed, weighed and stored for subsequent protein and RNA extractions or for histological analyses. All surgical procedures used in this study, were performed each scheduled day in an identical manner, following an *a priori* defined protocol in both technique and timing. Thus for plasma analyses, blood draws were taken at approximately the same time of day for dams and fetuses. Maternal blood was taken during c-section from the femoral artery, and fetal blood samples were taken from the abdominal aorta during necropsy.

Fetal studies were performed with 11 CTR, 7 HFD and 6 REV animals. Normal full-term pregnancies for Japanese macaques is 175 days, thus G130 is in the early 3^rd^ trimester. G130 was chosen after preliminary studies determined that this gestational age represented a critical period for the development of several metabolic systems: 1) hypothalamic circuits have started to develop, 2) there is widespread pancreatic β-cell development, 3) there is a full functioning placenta that is not at near term, and 4) there is very little white adipose tissue (WAT).

Previous work with this model has demonstrated significant increases in maternal leptin levels, decreases in maternal insulin sensitivity, and increases in maternal weight gain starting with the second year of maternal HFD exposure, and these changes persist and become greater through year four [47]. For our fetal studies, we are reporting differences in HFD fetuses whose mothers were exposed to the maternal diet for at least four consecutive years. In the fifth year of our studies, a diet-reversal protocol (REV) was initiated to assess dietary impact independent of maternal obesity. This protocol entailed switching a subgroup of adult females that had been exposed to a high-fat diet for four consecutive years, to a control diet 1–3 months before becoming pregnant and throughout the pregnancy.

Maternal breast milk for fatty acid analysis was obtained at postnatal day 30 (postnatal day 29.5±3 days, (mean ± SD)) from 6 CTR and 16 HFD dams giving birth to full term infants. The breast milk was collected during routine postnatal day 30 dual-emission X-ray absorptiometry (DEXA) procedures for the offspring. The DEXA procedure followed an *a priori* defined protocol and breast milk was obtained at approximately the same time of day for each animal. At 9:00 am, the mother was sedated with ketamine (15 mg/kg) or telazol, if ketamine resistant. The baby was separated from the mother for DEXA scanning. Two hours later, 0.5 mL oxytocin was injected intravenously into the mother to stimulate milk let down. The breast and nipple were massaged and milk was collected into a 15 mL conical vial. The milk sample was immediately stored on ice until centrifugation to separate aqueous milk and cream layers.

Due to study design parameters in which the focus of the REV group was on fetal effects, breast milk from the REV dams was not available. In addition, during the two-year period that the breast milk study encompassed, there were fewer pregnancies in CTR dams (15) than in the HFD dams (23). The volume of milk collected was highly variable and dependent on whether nursing had occurred immediately prior to our procedures. Visual inspection of the breast-milk was also used to identify and exclude samples that were discolored or contaminated with maternal blood. Thus, to obtain enough breast milk for the insulin, total protein, leptin and cytokine assays we performed, and to provide sufficient power for analysis, we sampled additional lactating CTR Japanese macaque dams. Milking of these additional CTR dams was performed during bi-annual colony health examinations and included milk that was older than 30 days post-partum (post-natal day 160±34 (mean ± SEM)). No differences were observed in the CTR group between the older milk and the 30 days post-partum milk for the insulin (12 CTR, 18 HFD dams) total protein (11 CTR, 8 HFD dams), leptin (20 CTR, 13 HFD dams) and cytokine assays (14 CTR, 18 HFD dams), so they were grouped for analysis. Detailed protocols for the insulin, total protein, leptin and cytokine assays are described in **Methods S1b**.

### Fatty acid profiles

The fatty acids present in each maternal diet, fasting maternal plasma breast milk and fetal plasma were analyzed by a modification of the methods described by Langerstedt et al. [54]. Deuterated fatty acids including d3C10:0, d3C14:0, d3C16:0, d3C18:0, d3C20:0 and d4C22:0 were added to samples prior to extraction as internal standards. Following hydrolysis and extraction, fatty acids were derivatized to the pentafluorobenzyl (PFB)-esters. The fatty acid-esters were analyzed by gas chromatography-mass spectroscopy (GC-MS) on a Trace DSQ (Thermoelectron) operating in the negative ion chemical ionization mode with methane as the reagent gas. The fatty acid-PFB esters were separated on a DB-5 ms capillary column (30 m×0.25 mm×0.25 µm) with helium as the carrier gas at a flow rate of 1 ml/min. Individual fatty acids were monitored with selected ion monitoring and a dwell time of 50 ms for each ion species. Each fatty acid was matched to the deuterated internal standard closest in length and retention time. Peak area ratios of known amounts of standard fatty acids and the internal standards were used to generate calibration curves to quantify unknowns using Xcalibur software.

### Dynamic range and efficiency curves for Real-Time PCR

Macaque specific primer sets were evaluated to determine the efficiency of our primer sets within a working range of cDNA concentrations and identify an optimum concentration of cDNA to use in Real-time PCR assays. A cDNA dilution series was made from four random samples from each dietary group. The cDNA was diluted based on initial RNA concentration and the assumption of 100% reverse transcription efficiency. The dilution series was designed so that each primer set started at an upper limit of 50 ng total cDNA/reaction and decreased in 10 ng increments to a minimum of 1 ng/reaction [55].

Primer validation Real-Time PCR reactions were run on an Applied Biosystems 7300 as relative quantification plates with SYBR master mix used at a 2× dilution. Following automatic thresholding and standard baseline adjustments after each run, Ct values were plotted as a function of the log (10) of the cDNA concentration and a linear slope was calculated [56]. Efficiency was calculated as 10∧(−1/slope) and was used for our experimental quantification [55]. For experimental assays we chose a cDNA concentration that gave us Ct threshold values across all dietary groups of between 20 and 32 cycles. In cases where we could not detect the target of interest in any diet group; threshold Ct values >35, or SYBR fluorescence not rising above background at all, we confirmed the presence of the target and the specificity of our target primers on fetal spleen processed in an identical manner as our liver samples. Additional protocols describing liver tissue RNA extractions (**Methods S1c**) and primer design (**Methods S1d**) are included as supporting information.

### Real-Time PCR

Experimental Real-Time PCR reactions were run on an Applied Biosystems 7300 as relative quantification plates. Target and endogenous control primers were used at a final concentration of 471 nM in a 21 µl reaction. SYBR master mix was used at the manufacturer's recommended 2× dilution. Dissociation curves were produced for every well to monitor primer amplification of a single target. Alg9 was used as an endogenous control for all our experiments. The Alg9 primer set was designed by core facilities at the Oregon National Regional Primate Center as an endogenous control for macaque gene expression analysis, and subjected to extensive gene stability validation in our model by use of the geNorm VBA applet [57], [58]. Primer sequences used for Real-Time PCR are described in **Table S1**.

Relative quantification of target gene expression was calculated across each dietary group using empirically derived efficiency values for each primer set and calculating an efficiency-corrected fold by the following formula:
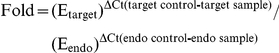
where E is the respective primer efficiency. The ΔCt_target_ was calculated by choosing one calibrator sample from the control diet group and subtracting subsequent target Ct values from that calibrator across all groups. In addition, ΔCt_endo_ was calculated by using endogenous control Ct values for the same calibrator sample as above, and subtracting subsequent endo Ct values from that calibrator [55]. In situations where the optimum cDNA concentration of our endogenous control differed from our target, we produced cDNA dilutions for the endogenous control and target from the same reverse-transcriptase reaction. Following the Real-Time PCR reaction random target well reactions from each diet group were run on a 2% agarose gel to verify amplicon singularity and size. Bands of the expected size were excised, gel purified (Qiaquick gel extraction kit, Qiagen #28706) and sequenced. Target specificity was confirmed by BLAST and comparing amplicon sequence with the NCBI macaque database.

### TUNEL assay

We used an ApopTag® Peroxidase *In-Situ* TdT end-labeling apoptosis detection kit (Chemicon S7100) on fetal CTR, HFD, and REV paraffin embedded fetal liver sections (Right lobe, 5 microns thick) as per manufacturers instructions. Tissue was deparaffinized in 3 washes of xylene, followed by graded alcohol (100%, 95% and 70%) rehydration. Proteinase K (20 ug/ml) digestion for 15 min at room temperature was followed by 2 washes in ddH_2_O. Endogenous peroxidases were quenched for 5 minutes in 3.0% hydrogen peroxide in 1× PBS. Following application of proprietary equilibration buffer, the TdT enzyme was incubated for one hour at 37°C. The TdT reaction was stopped by immersion into wash buffer and the anti-digoxigenin conjugate was applied and incubated at room temperature for 30 minutes. Following 4 washes in 1× PBS, the peroxidase substrate was developed for 6 minutes at room temperature. Samples were then washed in ddH_2_O and counterstained with methyl green.

### TUNEL imaging and quantification

Imaging was performed on a Marianas Digital Imaging workstation equipped with a Zeiss Axiovert 200 M inverted microscope (Zeiss Microimaging, Thornwood, NY) and a Coolsnap camera (Roper Scientific, Tucson, AZ) by a blinded observer. A montage of each liver section was created with the Marianas Digital Imaging workstation using a 2× objective. Stereological analysis of the 2× montage was performed by masking the hepatic area and placing 500 um×500 um regions with spacings of 1000 um×1000 um and a random offset of 69.4 (x-coordinate) and 513.4 (y-coordinate), on the image (SlideBook, Intelligent Imaging Innovations, Denver, CO). Coordinates of each region were recorded and then each region was imaged using a 10× objective. Following acquisition of between 20 to 40 10× images per section, masking was used to threshold and calculate the total hepatic parenchyma area. An additional mask was used to threshold TUNEL positive cells. Within the stereology program we excluded TUNEL positive debris under 5 microns in width.

Following these adjustments, the number of TUNEL positive stained cells (“events”) was automatically counted and the total hepatic area was recorded. The number of events was converted to a rate, defined as rate = (# events + 0.5)/ hepatic area, with the overall rate for each animal being summarized as the median rate among all animal-specific measurements. These rates were then log transformed (base 10) for analysis, with the log transformation aiding to stabilize variance and make the distribution of rates more symmetric. (The addition of 0.5 in the initial rate calculation was necessary to avoid taking the log of zero; among the 646 measurement only 7.6% were 0 counts). Analysis of variance was then used to determine whether the median rate differed according to diet.

Other analyses based on summarizing individual measurements in terms of mean rate, as well as non-parametric analysis (Kruskal-Wallis) applied to the current median-rate summary led to similar conclusions and are not reported. Similarly, a linear mixed effect model with count distributed according to a Poisson distribution (and area treated as an offset) found similar conclusions to our earlier and more simple approaches based on all summarizing forms (median/mean) of the log transformed rates; consequently, we present only results of the simpler analysis. All analyses performed using R version 2.6.1 (R Development Core Team (2007), R Foundation for Statistical Computing, Vienna, Austria. ISBN 3-900051-07-0, URL: http://www.R-project.org.) Graphs were produced with Prism software (GraphPad Software, Inc., La Jolla, CA).

### Data analysis

Data for all analyses not explicitly described above, were first compiled and tested for normality by Shapiro-Wilk with STATA (College Station, Texas) statistical software. Data were transformed to fit Gaussian distributions and tested for significance by ANOVA with a Bonferroni post-hoc analysis. Groups that did not attain a Gaussian distribution by transformation were first tested for overall significance by Kruskal-Wallis rank sum, followed by a Wilcoxon rank sum test with a Bonferroni adjusted alpha to determine significance between diet groups. Pair-wise analysis of the fatty acid association between each dam and their respective offspring was performed using pairwise correlation in STATA. We are reporting an overall Pearson correlation coefficient across our three diet groups for each analysis. In addition, maternal and fetal fatty acid samples were also tested by pairwise correlation in STATA for an overall association with maternal insulin resistance and glucose clearance. All graphs were made with Prism software (GraphPad Software, Inc., La Jolla, CA).

## Results

### Maternal diet nutritional analysis

To initiate our analysis of the impact that maternal HFD has on the developing NHP fetus, we chose to first examine differences in fatty acid composition between the CTR and HFD diets. Using gas chromatography-mass spectroscopy, we found that compared to the CTR diet, the maternal HFD has higher levels of myristic (C14:0), myristoleic (C14:1), palmitic (C16:0), palmitoleic (C16:1), stearic (C18:0), oleic (C18:1, n-9), linoleic (LA, C18:2, n-6), α-linolenic (ALA, C18:3, n-3), and arachidonic (AA, C20:4, n-6) fatty acids. In addition, the HFD had lower levels of eicosopentanoic acid (EPA, C20:5, n-3) and docosahexaenoic acid (DHA, C22:6, n-3) than the CTR diet. When fatty acid subtypes were combined into groups, the HFD contained much higher levels of total fatty acids, saturated, monounsaturated, polyunsaturated, essential fatty acids (EFA, sum of C18:2 and C18:3) and total n-6 fatty acids (sum of C18:2 and C20:4) than in the CTR diet. Total n-3 fatty acids (sum of C18:3, C20:5 and C22:6) were similar between HFD and CTR maternal diets; however, a greater than 2-fold increase in HFD total n-6 fatty acids resulted in an n-6∶n-3 ratio of 20∶1 compared to the 9∶1 n-6∶n-3 ratio in the CTR diet (**Table 1** **)**. Thus, the HFD has an n-6∶n-3 ratio that is reflective of current trends in the Western diet [59].

**Table 1:** Gas chromatography- mass spectrometry analysis of maternal chow.^1^

		DIET GROUP				
Fatty Acid	Common Name (Type)	CTR		HFD		
		Mean	SEM	Mean	SEM	HFD/CTR Ratio
C 14:0	Myristic	0.5	0.1	15.0	1.3	30
C 14:1	Myristoleic	0.004	0.001	0.4	0.1	100
C 16:0	Palmitic	6.1	2.0	24.7	5.7	4.1
C 16:1	Palmitoleic	0.2	0.0	3.4	0.5	17
C 18:0	Stearic	2.9	1.0	20.2	1.9	7
C 18:1	Oleic (N9)	9.5	2.1	52.9	15.4	5.6
C 18:2	Linoleic (N6)	18.9	5.0	40.6	14.6	2.1
C 18:3	Linolenic (N3)	0.5	0.1	1.2	0.4	2.4
C 20:4	Arachidonic (N6)	0.04	0.02	0.4	0.1	10
C 20:5	EPA (N3)	0.8	0.2	0.3	0.1	0.4
C 22:6	DHA (N3)	1.0	0.5	0.6	0.2	0.6
Total Fatty Acids		40.3	4.8	169.6	39.4	4.2
Total Saturated		9.4	3.0	59.9	8.8	6.4
Total Monounsaturated		9.7	2.0	56.6	15.9	5.8
Total Polyunsaturated		21.2	5.8	52.6	20.5	2.5
Total Essential Fatty Acids		19.4	5.1	41.8	15.0	2.2
Total N6		19.0	5.0	50.2	19.7	2.2
Total N3		2.2	0.8	2.4	0.8	0.9
N6∶N3 Ratio		8.8	1	19.9	0.9	2.3

1 All values are mean ± SEM and expressed as mg/g of dry chow.

### Maternal plasma lipid profiles

Lipid analysis of maternal plasma total fatty acids revealed that fasting levels of total fatty acids, total n-6, saturated, monounsaturated, polyunsaturated, and EFA's were not significantly different between the CTR, HFD and REV maternal diet groups (**Table 2**). However, total n-3 fatty acids were significantly reduced in the HFD group when compared to CTR and REV diet groups. Comprising the reduction of total n-3 fatty acids were significant decreases in EPA (n-3) and DHA (n-3) in the HFD group when compared to both the CTR and REV dietary groups. There was no change in DHA between CTR and REV; however, EPA was higher in the REV group when compared to CTR. There were no significant differences observed in α-linolenic acid (n-3) and linoleic (n-6) across the three dietary groups. A trend was observed for lower levels of arachidonic acid (n-6) in HFD maternal plasma, but this did not reach statistical significance (*p* = 0.06).

**Table 2:** Gas chromatography- mass spectrometry analysis of maternal plasma lipids.^1^

		DIET GROUP						
Fatty Acid	Common Name (Type)	CTR		HFD		REV		
		Mean	SEM	Mean	SEM	Mean	SEM	*p*-value^2^
C 14:0	Myristic	290.7	61.8	347.5	84.8	226.1	35.6	0.68
C 14:1	Myristoleic	4.7	1.0	3.3	0.4	4.7	0.9	0.52
C 16:0	Palmitic	2456.2	239.4	2187.7	209.8	2239.4	194.2	0.67
C 16:1	Palmitoleic	192.2	50.6	113.5	17.3	90.4	11.1	0.54
C 18:0	Stearic	796.7	60.1	1013.4	65.7	860.4	55.2	0.07
C 18:1	Oleic (N9)	828.7	129.0	1084.7	101.8	859.5	85.8	0.33
C 18:2	Linoleic (N6)	1167.9	150.6	1122.6	72.6	1395.6	231.8	0.73
C 18:3	Linolenic (N3)	27.8	4.0	28.6	2.1	31.3	4.5	0.81
C 20:4	Arachidonic (N6)	484.5	55.8	366.6	29.8	570.2	48.1	0.06
C 20:5	EPA (N3)	72.8^b^^,^ c	9.4	**5.2** a ^,^ c	0.8	122.0^a^^,^ b	13.1	**0.0001**
C 22:6	DHA (N3)	219.4^b^	33.8	**39.8** a ^,^ c	2.4	177.5^b^	21.2	**0.0001**
Total Fatty Acids		6541.5	632.3	6312.8	469.4	6377.7	568.6	0.9718
Total Saturated		3543.6	310.6	3548.6	310.8	3325.9	264.7	0.89
Total Monounsaturated		1025.5	170.5	1201.4	117.6	954.6	90.7	0.58
Total Polyunsaturated		1972.5	238.6	1562.8	81.8	2270.2	308.0	0.33
Total Essential Fatty Acids		1195.7	154.5	1151.2	74.7	1426.5	237.1	0.61
Total N6		1652.5	203.0	1489.2	80.3	1951.5	280.1	0.53
Total N3		320^b^	37.4	**73.7** a ^,^ c	2.7	330.8^b^	30.2	**0.0001**
N6∶N3 Ratio		5.2^b^	0.2	**20.2** a ^,^ c	0.9	6.1^b^	0.5	**0.0009**

1 All values are means ± SEM and are expressed as µmol/L. (n = 11 for CTR, n = 6 for HFD, n = 6 for REV).

2 Overall significance as determined by ANOVA or Kruskal-Wallis rank sum test.

a Significantly different from CTR, p<.0167, Bonferroni adjusted α.

b Significantly different from HFD, p<.0167, Bonferroni adjusted α.

c Significantly different from REV, p<.0167, Bonferroni adjusted α.

No significant changes were observed in total circulating n-6 fatty acids between the three dietary groups. However, decreases in EPA and DHA in the HFD group were large enough to significantly lower total n-3 levels in maternal plasma. Consequently, the HFD maternal plasma had a significantly higher n-6∶n3 ratio (20∶1) when compared to CTR (5∶1) or REV plasma (6∶1).

### Fetal plasma lipid profiles

Lipid analysis of fetal plasma also revealed that total fatty acids, saturated, EFA's and total n-6 fatty acid levels were not significantly different between the CTR, HFD and REV diet groups (**Table 3**). A significant increase in total polyunsaturated fatty acids was found in the REV group when compared to the HFD group but no statistical differences were observed when the CTR group was compared to either the HFD or REV diet groups. An increase in total monounsaturated fatty acids was observed in the HFD fetal plasma compared to the CTR and REV groups. The increase in monounsaturated fatty acids in the HFD group was due to higher levels of the major monounsaturated, oleic acid (C18:1, n-9), but the differences in oleic acid only reached statistical significance when the HFD was compared to the REV diet group.

**Table 3:** Gas chromatography- mass spectrometry analysis of fetal plasma lipids.^1^

		DIET GROUP						
Fatty Acid	Common Name (Type)	CTR		HFD		REV		
		Mean	SEM	Mean	SEM	Mean	SEM	*p*-value^2^
C 14:0	Myristic	125.1	18.2	164.0	34.5	127.2	32.9	0.57
C 14:1	Myristoleic	4.4	1.0	6.3	1.9	5.5	1.9	0.8
C 16:0	Palmitic	708.3	108.3	647.5	98.4	737.4	146.3	0.88
C 16:1	Palmitoleic	146.1	19.7	118.8	10.7	115.2	15.6	0.49
C 18:0	Stearic	455.0	37.7	419.1	63.9	385.7	22.3	0.6
C 18:1	Oleic (N9)	473.2	56.5	**724.7** c	73.9	390.2^b^	103.1	**0.02**
C 18:2	Linoleic (N6)	498.6	73.0	450.0	31.9	976.9	293.5	0.17
C 18:3	Linolenic (N3)	13.9	1.8	13.7	1.5	14.2	1.9	0.98
C 20:4	Arachidonic (N6)	572.6	152.4	417.3	111.7	675.6	229.3	0.4
C 20:5	EPA (N3)	42.6	9.1	30.9	5.4	67.9	20.3	0.29
C 22:6	DHA (N3)	273.9^b^	78.7	**52.2** a	6.0	259.4	119.3	**0.01**
Total Fatty Acids		3313.7	404.9	3044.4	243.2	3755.1	566.3	0.65
Total Saturated		1288.4	134.8	1230.5	175.4	1250.2	164.9	0.96
Total Monounsaturated		623.7	70.5	**849.8**	82.1	510.8	111.7	**0.05**
Total Polyunsaturated		1401.7	279.8	964.1^c^	118.7	**1994.0** b	372.4	**0.047**
Total Essential Fatty Acids		512.5	74.8	463.7	32.4	991.1	294.3	0.19
Total N6		1071.2	201.1	867.3	114.1	1652.5	310.9	0.09
Total N3		330.5^b^	80.1	**96.8** a,c	6.8	341.5^b^	110.1	**0.001**
N6∶N3 Ratio		3.6^b^	0.2	**8.9** a	0.9	6.7	1.7	**0.007**

1 All values are means ± SEM and are expressed as µmol/L. (n = 11 for CTR, n = 7 for HFD, n = 6 for REV).

2 Overall significance as determined by ANOVA or Kruskal-Wallis rank sum test.

a Significantly different from CTR, p<.0167, Bonferroni adjusted α.

b Significantly different from HFD, p<.0167, Bonferroni adjusted α.

c Significantly different from REV, p<.0167, Bonferroni adjusted α.

As observed in the maternal circulation, total n-3 fatty acids were significantly lower in the HFD group when compared to CTR and REV. This decrease was due to significantly lower levels of DHA in the HFD fetal plasma when compared to CTR and REV. Again, due to the decreases in HFD circulating total n-3 fatty acids, a significant increase in the n-6∶n-3 ratio was found between CTR (4∶1) and the HFD group (9∶1). In the REV diet group DHA and total n-3 fatty acids were normalized to CTR levels. However, due to an increase in REV plasma n-6 fatty acids, which was not itself significantly higher when compared to the CTR and HFD diet groups, the REV n-6∶n-3 ratio (7∶1) was only partially normalized to the CTR ratio. Thus, the reduced levels of n-3 fatty acids and elevated n-6∶n-3 fatty acid ratio found in the HFD maternal plasma were also observed in the HFD fetal plasma.

### Plasma n-6∶n-3 ratios are correlated between maternal and fetal circulation

The fasting levels of total fatty acids, saturated, monounsaturated and n-6 fatty acids are not different in the maternal plasma between dietary groups. Except for a small but significant increase in monounsaturated fatty acids, these findings are also observed in fetal plasma. However, we observed significant decreases in DHA and total n-3 fatty acids in both maternal and fetal HFD circulation that were normalized with maternal diet reversal. These findings suggest that circulating n-3 fatty acids, as essential fatty acids, are dependent on dietary supply and availability in both mother and fetus across our three dietary groups.

**Figure 1:**
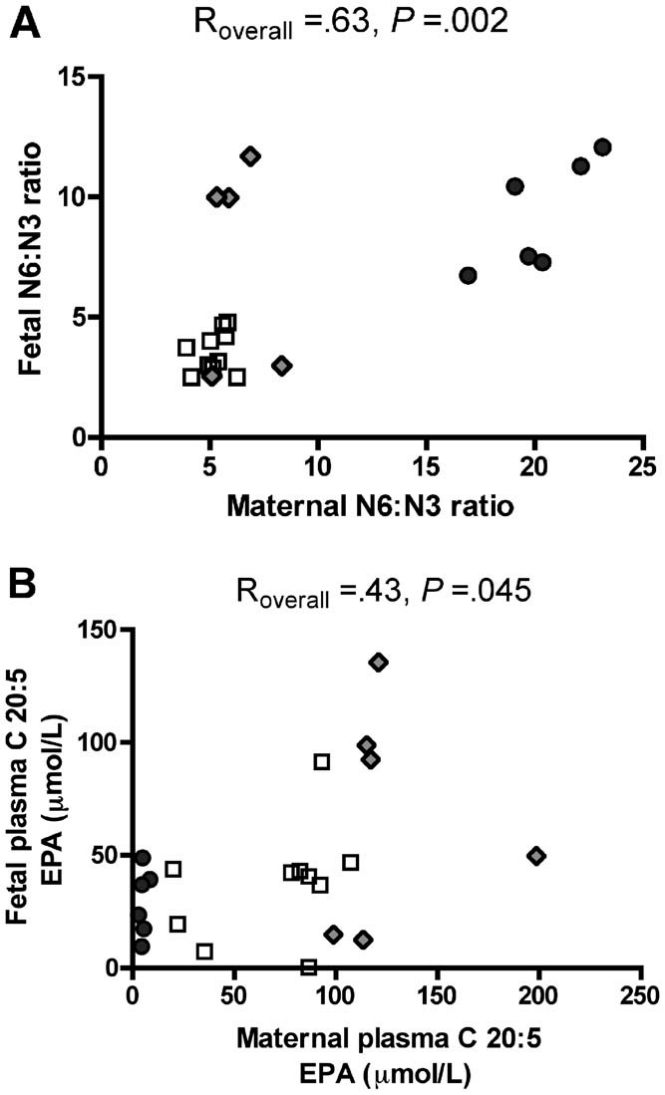
Correlation of fetal plasma fatty acids with maternal plasma fatty acids. Pair-wise correlation analysis of the plasma N6∶N3 fatty acid ratio (A), and plasma EPA levels (B), between CTR, HFD and REV Japanese macaque dams and their respective third trimester fetuses (*n* = 22 maternal/fetal pairs). Both the plasma N6∶N3 FA ratio and plasma EPA levels are correlated between maternal and fetal circulation. (CTR: white squares, HFD: dark grey circles, REV: grey diamonds).

Our model provides us with the ability to perform pair-wise analysis of circulating lipids between individual dams and their offspring to investigate associations between maternal and fetal parameters. Thus, we performed a pairwise correlation analysis and found that there was a significant correlation (**Figure 1A**) between the maternal n-6∶n-3 ratio and the n-6∶n-3 ratio found in the fetus (R_overall_ = .63, *p* = .002). In addition, we found a weaker but statistically significant association (**Figure 1B**) between maternal EPA and fetal EPA (R = .43_overall_, *p* = .045). However, we did not find a statistically significant association between maternal and fetal plasma DHA.

### Maternal obesity versus maternal consumption of a high-fat diet

Previous work with this model has shown that following chronic maternal consumption of a HFD two maternal phenotypes emerge [47]. Compared to CTR animals, diet-sensitive HFD dams are obese, hyperleptinemic, and insulin resistant. In contrast, the diet-resistant HFD dams had normal insulin secretion during glucose tolerance tests, similar body weights, body fat and circulating leptin levels relative to the maternal CTR diet group even after four years on the high-fat diet. To date, the majority of findings regarding fetal development with this model have been independent of maternal obesity and diabetes.

We tested whether maternal sensitivity to the HFD is associated with either maternal or fetal fatty acid levels by pairwise correlation. We found that the maternal insulin secretion (insulin AUC) and maternal glucose clearance (glucose AUC), as determined by third trimester maternal i.v. glucose tolerance testing, were not correlated with fasting plasma levels of any maternal fatty acids acquired at time of cesarean section (G130).

**Figure 2:**
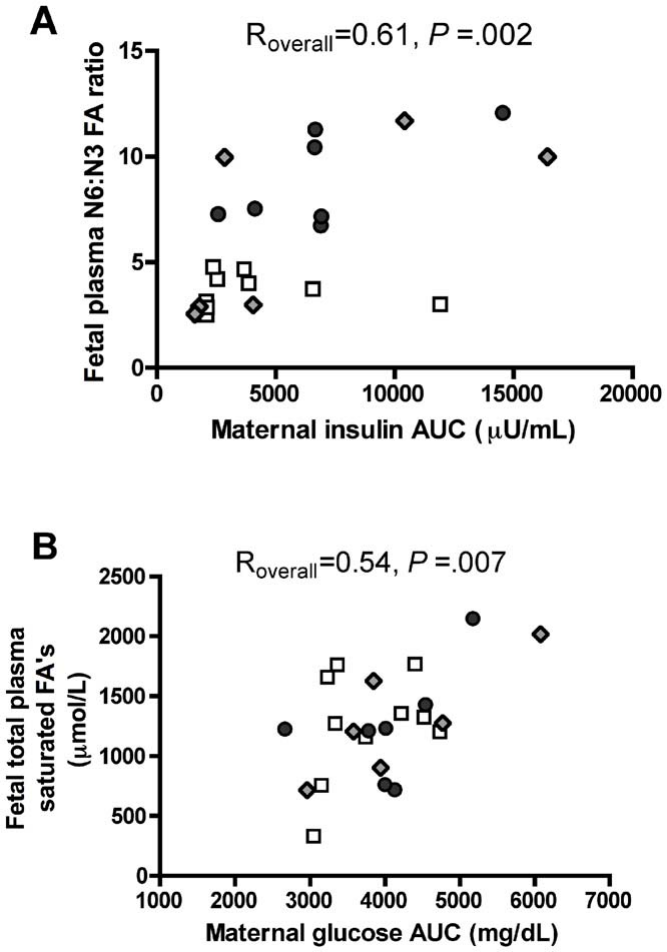
Correlation of fetal plasma fatty acids with maternal insulin resistance and glucose clearance. Pair-wise correlation analysis of fetal plasma N6∶N3 fatty acid ratio with respective maternal insulin AUC (A), across CTR, HFD and REV maternal diet groups. Pair-wise correlation analysis of total fetal plasma saturated FA's with respective maternal glucose AUC (B), across CTR, HFD and REV maternal diet groups. (*n* = 23 maternal/fetal pairs). Fetal N6∶N3 ratio is positively correlated with maternal insulin AUC. Total fetal plasma saturated FA's are correlated with maternal glucose AUC. (CTR: white squares, HFD: dark grey circles, REV: grey diamonds).

Interestingly, we found that the fetal plasma n-6∶n-3 ratio was positively correlated with maternal insulin AUC (R_overall_ = .61, *p* = .002, **Figure 2A**). No other fetal plasma fatty acids assayed were correlated with the maternal insulin AUC. Additionally, we found that the total fetal plasma levels of saturated fatty acids (R_overall_ = .54, *p* = .007, **Figure 2B**) were correlated with the maternal glucose AUC, as were the individual saturated fatty acids C14:0 (myristic acid; R_overall_ = .43, *p* = .04), C16:0 (palmitic acid; R_overall_ = .45, *p* = .04). In addition, C18:1 (oleic acid; R_overall_ = .43, *p* = .04) and C18:3 (α-linolenic acid ( n-3); R_overall_ = .50, *p* = .01 ) were also positively correlated with maternal glucose AUC (**Figure S1**). These data extend previous associations found between maternal diet sensitivity and fetal outcomes in our model [53]. Our findings suggest that while maternal diet has been the primary predictor of fetal outcomes thus far, other maternal factors (e.g. obesity, hyperinsulinemia, etc.) may also play important roles in determining the fatty acid milieu of the developing fetus.

### Inflammation in the high-fat fetal liver

Recent work with this model suggested that non-alcoholic fatty liver disease (NAFLD) is present in the HFD fetal liver. McCurdy et al. demonstrated that oxidative damage, hepatic steatosis, and upregulation of phospho-JNK1 that was highly correlated with levels of fetal liver triglycerides were present in the HFD fetal livers [47]. To extend these findings, we investigated whether inflammation and consequent evidence of non-alcoholic steato-hepatitis (NASH) was present in the HFD fetal liver. We used Real-time PCR to evaluate the expression of inflammatory markers between CTR, HFD and REV fetal livers (**Table 4**). We found that the expression of interleukin-10 (IL-10) was significantly different across the three dietary groups and lower in the REV group, but post hoc tests did not show statistical significance when compared to the CTR and HFD diet groups. Unexpectedly, the expression of Arginase-1 in the REV diet group, a marker of Th_2_ macrophage activation [60], [61], was significantly decreased when compared to the CTR and HFD groups. No differences were observed in the expression of any of the other inflammatory markers we assayed between the CTR, HFD and REV maternal diet groups. These data suggest that the fetal liver is not the origin of the increased levels of pro-inflammatory cytokines found in fetal circulation in previous work with this model [47].

**Table 4:** Inflammatory marker mRNA expression in fetal liver.^1^

	DIET GROUP						
Target	CTR		HFD		REV		*p*-value^2^
	Mean	SEM	Mean	SEM	Mean	SEM	
Interferon-γ	0.42	0.11	0.38	0.11	0.32	0.06	0.87
Interleukin-1β	0.95	0.26	1.05	0.24	0.89	0.11	0.82
Interleukin-4	undetected		undetected		undetected		n/a
Interleukin-6	undetected		undetected		undetected		n/a
Interleukin-10	0.97	0.06	1.19	0.18	0.69	0.11	**0.045**
I-TAC (CXCL11)	0.93	0.12	1.06	0.08	0.82	0.15	0.1
Lymphotoxin-α	0.76	0.11	0.79	0.08	0.77	0.05	0.59
MCP-1 (CCL2)	0.78	0.1	0.81	0.14	0.75	0.06	0.91
Tumor Necrosis Factor-α	0.82	0.16	0.71	0.07	0.53	0.08	0.28
Arginase-1	1.01	0.09	1.00	0.07	**65** a ^,^ b	0.04	**0.009**
C-Reactive protein	1.93	0.32	1.48	0.22	2.19	0.41	0.11

1 All values are means ± SEM and are expressed as relative fold to CTR calibrator sample. (n = 7 for CTR, n = 8 for HFD, n = 7 for REV).

2 Overall significance as determined by Kruskal-Wallis rank sum test.

a Significantly different from CTR, p<.0167, Bonferroni adjusted α.

b Significantly different from HFD, p<.0167, Bonferroni adjusted α.

c Significantly different from REV, p<.0167, Bonferroni adjusted α.

### Apoptosis in high-fat fetal liver

**Figure 3:**
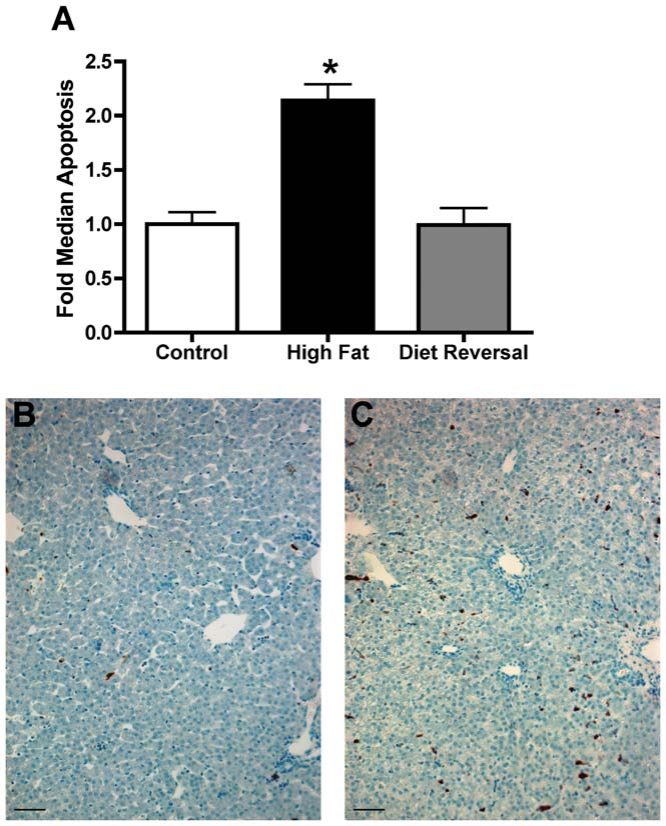
Fetal hepatic apoptosis. Quantification of TUNEL positive cells normalized to hepatic parenchyma area in G130 macaque fetal liver for CTR, HFD, and REV maternal diet groups (A). Data are expressed as fold increase over CTR of the median rate ± standard error (CTR; n = 6, HFD; n = 7, REV; n = 6, ^*^ *P*<.05 versus CTR, ANOVA). Representative brightfield images for TUNEL staining in CTR (B) and HFD (C) G130 fetal liver. TUNEL staining was returned to CTR levels following maternal diet reversal (REV), thus representative REV image is not pictured. Scale bar, 50µm.

During gestation the fetal liver directly receives about 50% of maternal blood flow via the branch of the umbilical vein that connects to the portal vein [62]. Thus, the fetal liver, and particularly the right lobe of the fetal liver, is anatomically positioned to be directly affected by factors that are present in the umbilical circulation. Given the increases in pro-inflammatory cytokines found in the fetal umbilical circulation and the evidence of NAFLD reported in previous work [47], we performed a TUNEL assay to examine whether evidence of increased hepatic apoptosis was present in fetal livers exposed to a maternal HFD. We found that there is a significant increase in the number of apoptotic cells in the HFD fetal liver compared to CTR when normalized to hepatic area (2.14 fold increase, *p*<.05). Importantly, by switching a subgroup of HFD mothers to a control diet during a subsequent pregnancy (REV), apoptosis in the fetal liver was completely normalized to baseline (**Figure 3**).

### Postnatal Studies

To begin to understand the long-term metabolic programming effects that a maternal high-fat diet has on the offspring, it is necessary to separate effects that occur *in-utero* from changes that occur after parturition. Lactation is a critical period of development for the offspring that may be sensitive to maternal obesity and consumption of a high-fat diet. To begin to address the effects that maternal obesity and overnutrition may have during lactation, we performed gas chromatograph mass spectroscopy on maternal breast milk to characterize postnatal exposure of the offspring to maternal fatty acids. In addition, we assayed levels of insulin, total protein, and leptin as well as interleukin-1β present in maternal breast milk.

### Maternal breast milk lipid profiles

In the breast milk we found no changes in total fatty acids in the HFD group versus CTR. C14:0 (myristic), C16:0 (palmitic), C18:0 (stearic) and total saturated fatty acids were not higher in the HFD group when compared to control (**Table 5**). There were no differences in total levels of monounsaturated fatty acids as well as C16:1 (palmitoleic) and C18:1 (oleic). Total polyunsaturated fatty acids were unchanged in HFD breast milk when compared to CTR. Total n-6 fatty acids, as well as C18:2 (linoleic) and C20:4 (arachidonic), were unchanged in the HFD breast milk when compared to CTR. Total n-3 fatty acids were also unchanged between the CTR and HFD diet groups. However, C20:5 (EPA, n-3) and C22:6 (DHA, n-3) were significantly lower in the HFD breast milk when compared to CTR breast milk. C18:3 (linolenic, n-3) was the largest component of total n-3 fatty acids assayed and was unchanged between the CTR and HFD diet groups. Thus, the observed decreases in EPA and DHA were not large enough to significantly lower the total levels of breast milk n-3 fatty acids. The mean n-6∶n-3 ratio was higher in the HFD (19∶1) breast milk than CTR breast milk (9∶1). However, a large variance in both groups prevented the increased HFD n-6∶n-3 ratio from attaining significance. Overall, the significant decreases in EPA and DHA in HFD breast milk reflect what was also observed in maternal and fetal plasma.

**Table 5:** Gas chromatography- mass spectrometry analysis of maternal breast milk lipids.^1^

		DIET GROUP				
Fatty Acid	Common Name (Type)	CTR		HFD		*p*-value^2^
		Mean	SEM	Mean	SEM	
C 14:0	Myristic	25.8	16.1	49.8	12.4	0.18
C 14:1	Myristoleic	24.6	11.3	33.7	11.7	0.51
C 16:0	Palmitic	60.1	18.7	87.7	13.3	0.42
C 16:1	Palmitoleic	64.0	24.0	52.4	17.1	0.42
C 18:0	Stearic	27.9	8.3	46.4	6.8	0.10
C 18:1	Oleic (N9)	43.2	23.7	67.5	15.5	0.18
C 18:2	Linoleic (N6)	64.6	21.5	56.1	5.5	0.51
C 18:3	Linolenic (N3)	13.2	5.0	15.4	5.3	0.61
C 20:4	Arachidonic (N6)	1.7	0.5	1.9	0.3	0.48
C 20:5	EPA (N3)	0.6	0.2	**0.2**	0.1	**0.012**
C 22:6	DHA (N3)	2.3	0.6	**0.3**	0.0	**0.0007**
Total Fatty Acids		328.1	61.5	411.6	32.5	0.30
Total Saturated		113.9	41.2	183.9	29.9	0.21
Total Monounsaturated		131.8	24.1	153.7	18.6	0.61
Total Polyunsaturated		82.4	20.2	74.0	7.0	0.71
Total Essential Fatty Acids		77.8	19.0	71.5	6.7	0.71
Total N6		66.3	21.9	58.0	5.7	0.51
Total N3		16.1	4.9	16.0	5.4	0.51
N6∶N3 Ratio		9.1	3.6	18.9	3.5	0.16

1 All values are means ± SEM and are expressed as µmol/L. (n = 6 for CTR, n = 16 for HFD).

2 Overall significance as determined by Student's T-test or Wilcoxon rank sum test.

**Figure 4:**
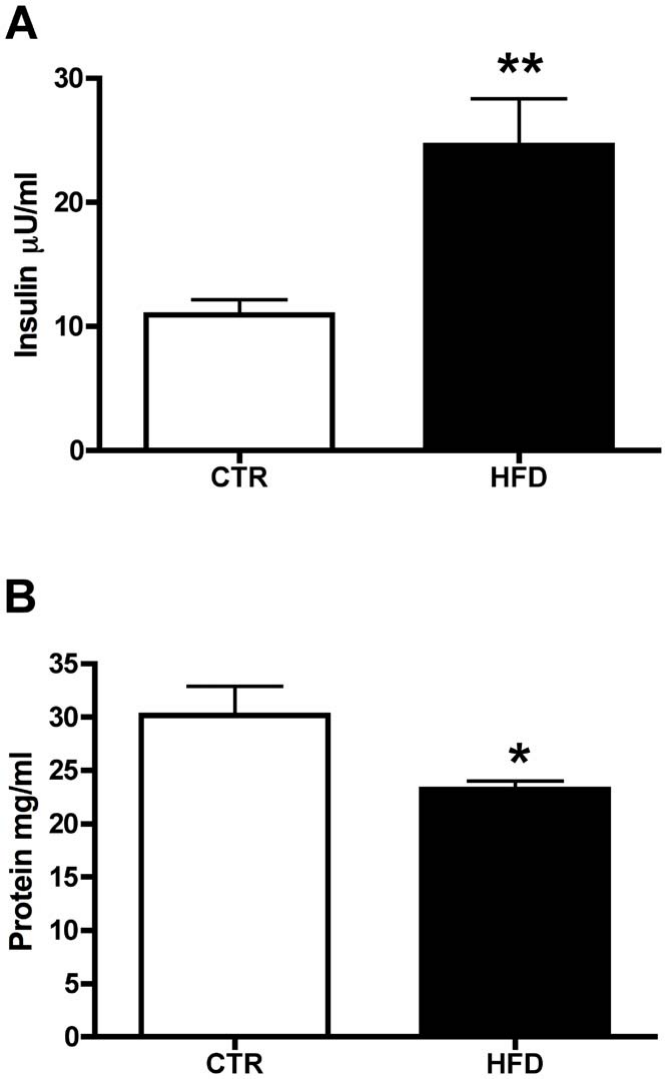
Maternal breast milk insulin and protein. Analysis of insulin (A) and total protein levels (B) in breast milk from macaque dams in CTR (white bars) and HFD (black bars) maternal diet groups. **A**. Insulin was assayed by a commercially available primate RIA kit. HFD dams have significantly higher levels of insulin in their breast milk than CTR dams ( CTR; *n* = 11, HFD; *n* = 17, ^**^ *P*<.01 versus CTR, Wilcoxon rank sum test). **B**. Macaque breast milk total protein levels were measured from the aqueous layer using a BCA™ Protein Assay kit across CTR (white bars) and HFD (black bars) maternal diet groups. HFD breast milk contains significantly lower levels of total protein when compared to CTR ( CTR; *n* = 13, HFD; *n* = 17, ^*^ *P*<.05 versus CTR, Student's t-test).

To further characterize the effects that maternal HFD had on breast milk, we performed radio-immunoassays for insulin and leptin. We found that insulin levels in maternal breast milk are significantly higher (2-fold) in HFD mothers versus CTR (**Figure 4A**). We found no changes in the levels of leptin in maternal breast milk between CTR and HFD, although the levels were quite low in both groups (data not shown). We also found no differences in the levels of the inflammatory cytokine IL-1β between CTR and HFD breast milk (data not shown). Total protein levels in HFD breast milk are significantly lower than in the CTR breast milk (**Figure 4B**).

### Postnatal phenotype

**Figure 5:**
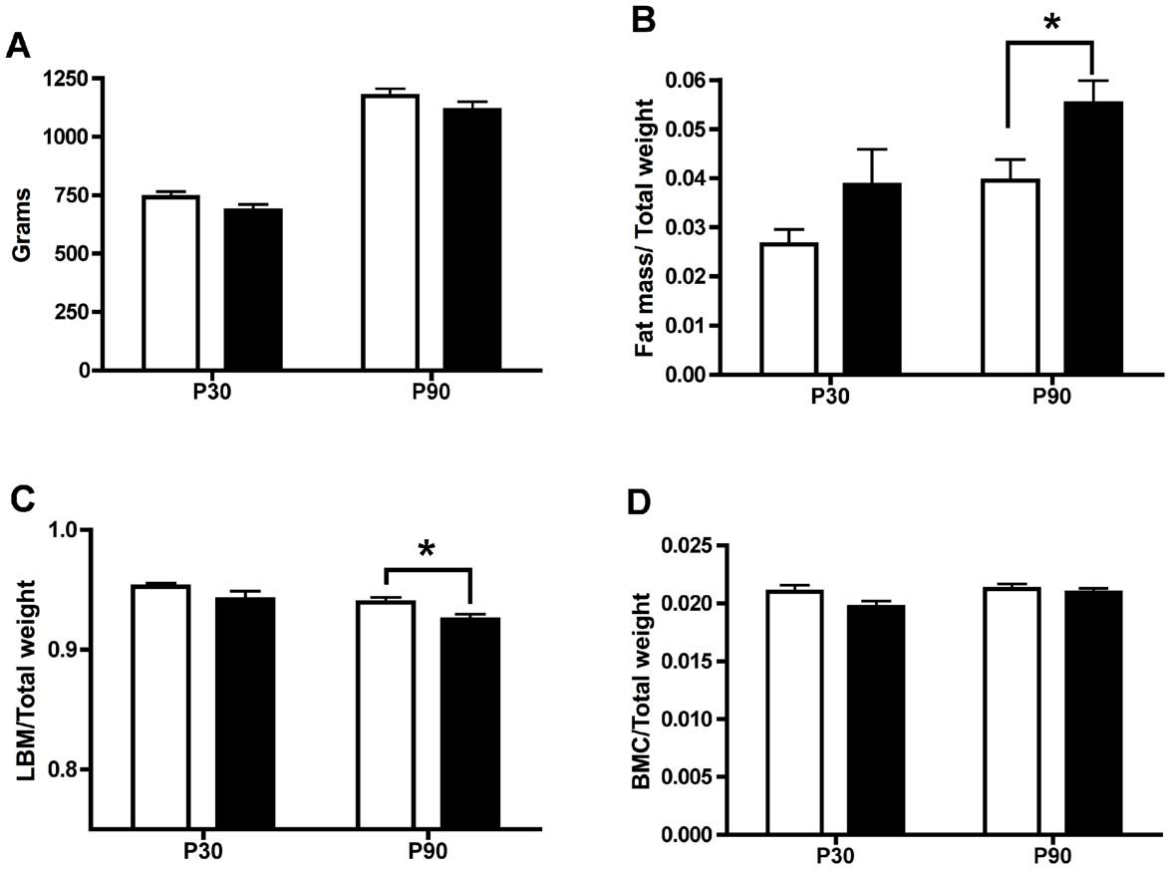
Offspring body composition. DEXA analysis of macaque offspring at post-natal day 30 (P30) and post-natal day 90 (P90) from CTR (white bars) and HFD (black bars) diet groups. **A**. Total weight of macaque offspring at P30 and P90. **B**. Normalized fat mass of macaque offspring between CTR and HFD diet groups at P30 and P90 (^*^ *P*<.05 versus CTR, Student's t-test). **C**. Normalized lean body mass (LBM) of macaque offspring between CTR and HFD diet groups at P30 and P90 (^*^ *P*<.05 versus CTR, Student's t-test). **D**. Normalized bone mineral content (BMC) of macaque offspring between CTR and HFD diet groups at P30 and P90. All data is expressed as mean ± standard error (P30 CTR; *n* = 15, P30 HFD; *n* = 17, P90 CTR; *n* = 13, P90 HFD; *n* = 19).

Given the proinflammatory environment our cohort of HFD animals were exposed to *in-utero*, combined with increased apoptosis in the fetal liver and the significant changes in breast milk composition, we examined the offspring from CTR and HFD dams to determine if phenotype differences were apparent in the postnatal period. Previous work with this model demonstrated that the offspring of HFD dams had similar bodyweights at postnatal day 30 (P30) and post-natal day 90 (P90) as CTR offspring, and higher levels of body-fat at P90 [47]. The current results again showed that body weights were similar between the CTR and HFD offspring at the P30 and P90 time points (**Figure 5A**) and that the HFD offspring had higher body fat at P90 than CTR offspring, as determined by DEXA scanning (**Figure 5B**). In addition, HFD offspring had significantly lower lean body mass than CTR offspring at P90 (**figure 5C**). We measured bone mineral content as well and found no differences at either P30 or P90 offspring between the two diet groups (**Figure 5D**). Thus, while total body weights are similar at P90 between CTR and HFD offspring, the HFD offspring have higher body fat and lower lean body mass than CTR offspring.

## Discussion

While much work has been done in the NHP to highlight the effects that maternal nutrient deprivation has on development of metabolic systems in the offspring [63]–[67], there is a fundamental gap in understanding the contributions that maternal obesity and maternal nutrient excess provide to metabolic programming. Previous work with this model has demonstrated that a maternal HFD altered fetal development and expression of key hypothalamic neuropeptides in the context of hypothalamic inflammation [49]. Furthermore, HFD fetal plasma contained elevated levels of inflammatory cytokines, and elevated triglycerides and glycerol that were highly correlated with maternal levels. In the liver, HFD fetuses had premature gluconeogenic gene expression, steatosis, elevated triglyceride content, and oxidative stress. The majority of these changes were observed irrespective of maternal obesity or maternal insulin resistance status and persisted into the early postnatal period. Importantly, switching HFD mothers to a control diet during pregnancy alone (diet-reversal; REV) normalized a number of the observed fetal hepatic pathologies towards control levels [47].

In the present study, we report in the nonhuman primate that chronic maternal HFD consumption, independent of maternal obesity or diabetes, leads to significantly reduced plasma levels of n-3 fatty acids in fasted HFD pregnant dams and third trimester fetuses. Our dietary model was designed to mimic the typical Western diet being consumed by a majority of pregnant women in the developed world. Whether the pathology is induced by elevated dietary fat content *per se* or is instead due to a change in dietary fatty acid composition (e.g. elevated n-6∶n-3 ratio) was not addressed by this experimental design. Furthermore, we cannot draw conclusions regarding whether specific fatty acid manipulations (e.g. n-3 supplementation) would have beneficial effects on the developing fetus.

In HFD dams we observed significantly reduced fasting plasma levels of DHA, EPA and total n-3 fatty acids. In the HFD fetal circulation, plasma levels of DHA and total n-3 fatty acids were also significantly reduced when compared to the CTR diet animals. HFD breast milk contained lower levels of EPA and DHA than CTR breast milk, however total n-3 fatty acids were not different between CTR and HFD breast milk. We also observed that apoptosis was significantly increased in the HFD fetal liver. Importantly, we found that returning HFD dams to a CTR diet during pregnancy normalized plasma n-3 fatty acids in pregnant dams and fetuses and returned fetal hepatic apoptosis to control levels.

The maternal HFD diet had by definition a much higher total fat content than the CTR diet including much higher levels of saturated, monounsaturated and polyunsaturated fats. Among the essential fatty acids, the HFD also contained slightly higher levels of linolenic acid (C18:3 n–3) but double the levels of the more abundant linoleic acid (C18:2 n–6) and total n-6 fatty acids. Consequently the HFD chow n-6∶n-3 ratio was 2-fold higher than the CTR n-6∶n-3 ratio. The high saturated and monounsaturated fatty acids in the HFD reflect that its fat largely came from animal sources (lard, animal fat, and butter), versus the CTR diet (grains and soybean oil). Also included in the maternal HFD were daily calorie-dense treats made from peanut butter.

In humans, a recent report showed that low-nutrient-density foods, consisting of refined carbohydrates and animal products high in saturated fat, were the major contributors to the total energy intake for a cohort of pregnant women [68]. While this study was small in scope, the consumption of a diet that is high in calories and saturated fats is similar to findings from nationally representative studies of children and non-pregnant women [69], [70]. Thus, our NHP HFD has strikingly similar characteristics to what is known about human dietary choices during pregnancy.

The present data are particularly relevant in light of the fact that over the last 100 years dietary n-6∶n-3 ratios have gone from being close to 1∶1 to approximately 20∶1 [71]. There is strong evidence suggesting that cellular membrane long chain polyunsaturated fatty acid composition is largely determined by dietary ratios [72]. Thus, the maternal and fetal plasma n-6∶n-3 ratio mirrored the n-6∶n-3 ratio of the diet in each diet group. The elevated n-6∶n-3 ratio found in the HFD chow was a result of increases in n-6 fatty acids. Notably, the maternal and fetal HFD plasma n-6∶n-3 ratio was driven by significant decreases in n-3 fatty acids when compared to CTR plasma. In particular, DHA was significantly decreased in both the maternal and fetal HFD circulation. Furthermore, significant decreases in EPA and DHA were observed in breast milk. Thus, the HFD offspring are provided with decreased levels of EPA, DHA and n-3 fatty acids during both fetal and early postnatal life, developmental periods that are dependent solely on maternal transfer of nutritional substrates for normal growth.

The n-3 and n-6 long chain polyunsaturated fatty acids, particularly DHA and AA, are critical for proper infant growth and neurodevelopment (reviewed in [73]). DHA and AA are both highly enriched in neural tissue while DHA is the major component of retinal photoreceptor membranes [74]–[76]. While only non-esterified fatty acids (NEFA) can be transferred from mother to fetus directly, other mechanisms, including hydrolysis of triglycerides and receptor mediated transfer, allow fatty acids including docosahexaenoic acid (DHA, 22:6 n-3), eicosapentaenoic acid (EPA, 20:5 n-3) and arachidonic acid (AA, 20:4 n-6), to be transferred through the placenta to the fetus [77]–[81].

In the human fetus, there is limited capacity for *de novo* lipogenesis and the precursors for fetal fat accretion are primarily supplied trans-placentally and consist of maternal substrates derived from lipids rather than from glucose [81]–[83]. It has also been shown in baboons that while the fetus has the capacity to synthesize DHA from its EFA precursor, α-linolenic acid, preformed maternal DHA is preferentially used for DHA accretion in the fetal brain [84]–[86]. Thus, the composition of the fatty acid supply to the fetus is mainly determined by maternal lipid profile and suggests that modifications of maternal diet or metabolic homeostasis will affect delivery of lipid substrates to the fetus [87], [88]. Our results support these findings and demonstrate that decreased circulating levels of DHA, total n-3 fatty acids, and an elevated n-6∶n-3 ratio was recapitulated in both maternal and fetal circulation.

Previous work in nonhuman primates demonstrated that dietary deprivation of n-3 fatty acids, and consequent decreases in plasma n-3 fatty acids during the prenatal and postnatal periods, had profound effects on brain and visual system fatty acid composition and retinal function of fetuses and infants [89], [90]. Decreased levels of n-3 fatty acids during development have also been associated with altered acetylcholine and dopamine release in rodents [91], [92]. Recently it was reported in our model, that fetuses exposed to a maternal HFD displayed significant changes in central serotonergic systems and nearly 78% of the HFD offspring displayed increased anxious or aggressive behavior during behavioral tests at postnatal day 130 [50]. Our work demonstrates that circulating levels of DHA and total n-3 fatty acids are significantly reduced in the HFD maternal and fetal plasma. Thus while the changes in fetal brain development previously reported in our model are likely to be multifactorial, our data suggests that the lower levels of plasma DHA and n-3 fatty acids found in the HFD fetal circulation may be partly responsible.

While the maternal HFD chow has decreased levels of EPA and DHA when compared to the CTR chow, the levels of total n-3 fatty acids were not different between two diets. In addition, the maternal HFD has much higher levels of α-linolenic acid (C18:3, n-3), an essential fatty acid precursor necessary for DHA synthesis, than the CTR chow. Neuringer et al. [90] demonstrated in the NHP that plasma levels of DHA could be maintained in pregnant dams fed a diet containing 8% α-linolenic acid despite undetectable levels of pre-formed DHA. It has been well established that the desaturases responsible for synthesis of DHA from α-linolenic acid are subject to regulation from dietary and hormonal factors [93]–[98]; in particular, n–3 and n–6 fatty acids compete as substrates for these desaturases as well as for uptake into tissues. Thus it is reasonable to conclude that the decreased levels of maternal plasma DHA and total n-3 fatty acids we observed are due to the high n–6∶n–3 ratio of the HFD.

The present study expands upon previous findings that suggest that maternal diet can lead to severe inflammatory and oxidative stress in the fetal liver. McCurdy et al. explored the effects of maternal HFD in this model and demonstrated evidence of fetal hepatic steatosis, oxidative stress, upregulation of heat-shock proteins, and increased phosphorylation of *c*-Jun NH_2_-terminal kinase (p-JNK), and increased inflammatory cytokines in the fetal circulation [47]. While our data does not show that the HFD fetal liver is the primary site of cytokine synthesis (at least at a transcriptional level), the circulating inflammatory insult to the developing fetal liver in our model is nonetheless quite significant.

Hepatocyte apoptosis is a marker of disease severity in numerous hepatic disease states [99]–[101]. In fact, the severity of hepatocyte apoptosis is significantly correlated with histopathological and biochemical markers of NASH and hepatic fibrosis [102]. There is evidence that there are connections between fatty acids, hepatic steatosis and hepatic apoptosis. For example, incubation of HepG2 cells *in-vitro* with saturated and monounsaturated fatty acids produced steatosis and p-JNK-dependent apoptosis that was more pronounced with saturated fatty acids [103]. In primary rat hepatocytes, treatment with oleic and stearic acid induced steatosis and sensitized hepatocytes to cytotoxicity mediated by tumor necrosis factor related apoptosis inducing ligand (TRAIL) [104]. Our data show a significant increase in the numbers of apoptotic cells in fetal livers exposed to a maternal high-fat diet. Previous findings of severe hepatic steatosis, increases in p-JNK, and high circulating levels of inflammatory cytokines [47], as well as our current findings of increased oleic acid in the fetal circulation are consistent with previously described mechanisms of hepatic apoptosis. To our knowledge, the increased apoptosis in the HFD fetal liver is a novel finding that reinforces the extent of damage occurring within the developing fetal liver. However, the regenerative capacity of the liver is formidable and studies already in progress will determine whether permanent hepatic damage is evident in these animals.

Our NHP model is a sophisticated and effective tool that makes it possible to quickly translate our findings into human clinical research studies. In addition to our findings, previous work with this model highlights the complex relationship between maternal diet and obesity. It has been shown that fetal serum metabolites are reduced under maternal high fat diet conditions. In agreement with our fatty acid findings, changes in specific fetal serum metabolites were associated with maternal diet, while others were associated with maternal obesity and insulin resistance [53]. Future studies are needed to untangle the contribution of maternal phenotype from maternal diet and their combined effects on fetal development before comprehensive interventions are employed. Within the midst of the present childhood obesity epidemic, it is critical that we move our findings forward into human studies and potentially into the realm of public health policy and clinical practice.

## Supporting Information

Figure S1 **Correlation of fetal plasma fatty acids with maternal glucose clearance.** Pair-wise correlation analysis of fetal plasma Myristic; C 14:0 (A), Palmitic; C 16:0 (B), Oleic; C 18:1 (C), and Linolenic; C 18:3 (D) FA's with respective maternal glucose AUC across CTR, HFD and REV maternal diet groups (*n* = 22–23 maternal/fetal pairs). (CTR: white squares, HFD: dark grey circles, REV: grey diamonds).
(TIF)

Table S1 **Macaque primer sequences.** Primer sequences used in this study for Real-time PCR amplification of macaque inflammatory markers. Genbank accession numbers are also provided. Definition of abbreviations: F, forward primer; R, reverse primer.
(DOC)

Methods S1 **Additional methods used in this study.**
(DOC)
