# Supplementary figures and images for: Maternal High Fat Diet Is Associated with Decreased Plasma n–3 Fatty Acids and Fetal Hepatic Apoptosis in Nonhuman Primates
Source: PLoS One. 2011 Feb 25;6(2):e17261. doi: 10.1371/journal.pone.0017261 (PMC3045408; doi:10.1371/journal.pone.0017261)

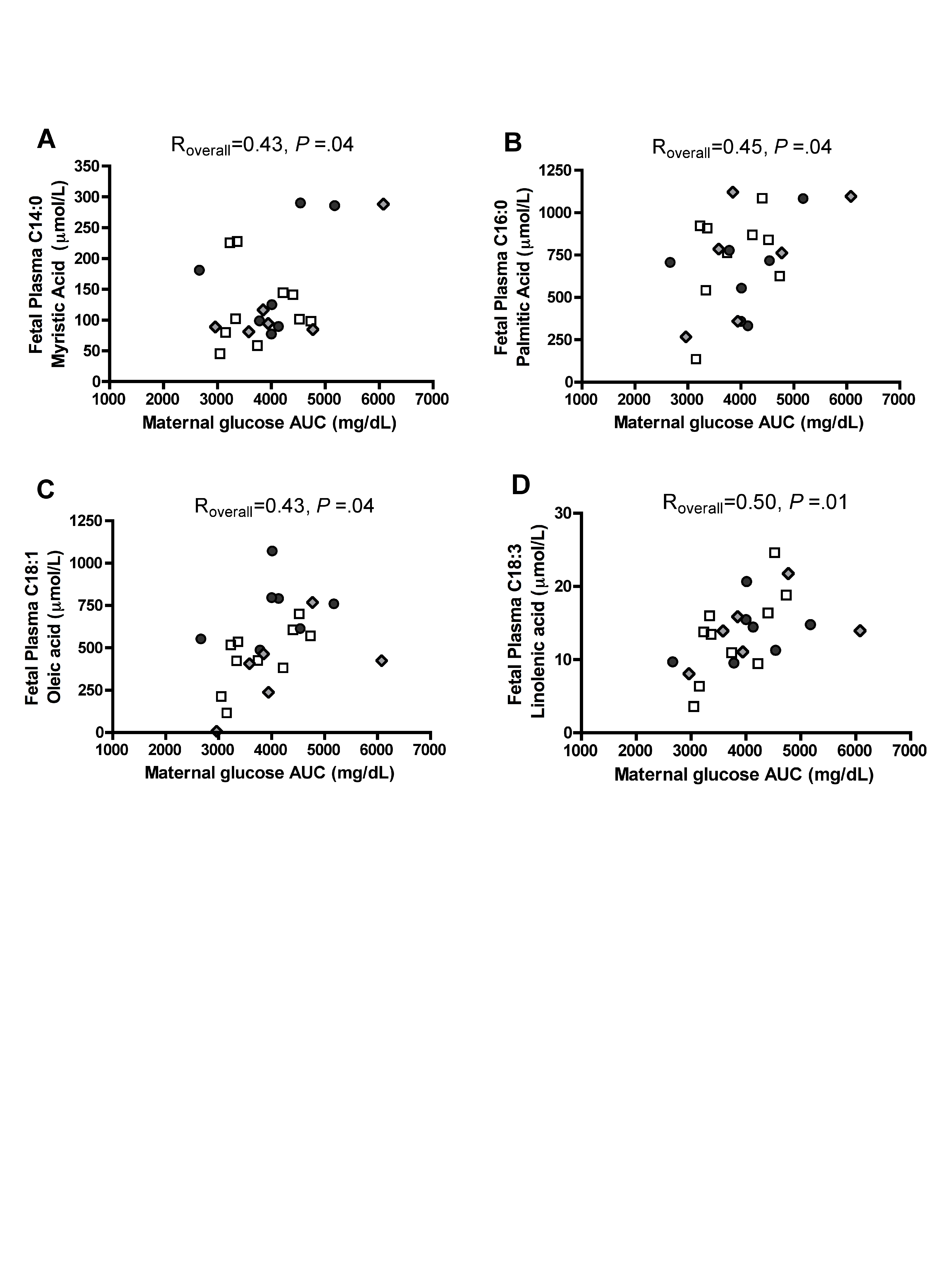

Supplement: Figure S1 — Correlation of fetal plasma fatty acids with maternal glucose clearance. Pair-wise correlation analysis of fetal plasma Myristic; C 14:0 (A), Palmitic; C 16:0 (B), Oleic; C 18:1 (C), and Linolenic; C 18:3 (D) FA's with respective maternal glucose AUC across CTR, HFD and REV maternal diet groups (n = 22–23 maternal/fetal pairs). (CTR: white squares, HFD: dark grey circles, REV: grey diamonds). (TIF) [file pone.0017261.s001.tif]
