# Supplementary material for: Maternal High Fat Diet Is Associated with Decreased Plasma n–3 Fatty Acids and Fetal Hepatic Apoptosis in Nonhuman Primates
Source: PLoS One. 2011 Feb 25;6(2):e17261. doi: 10.1371/journal.pone.0017261 (PMC3045408; doi:10.1371/journal.pone.0017261)
Supplement: Table S1 — Macaque primer sequences. Primer sequences used in this study for Real-time PCR amplification of macaque inflammatory markers. Genbank accession numbers are also provided. Definition of abbreviations: F, forward primer; R, reverse primer. (DOC) [file pone.0017261.s002.doc]

**TABLE S1.**

Primer sequences for Real-time PCR amplification of macaque inflammatory markers.

Genbank

mRNA Sequence (5′-3′) Accession No.

IFNG F: TGTCCAACGCAAAGCAGTACA NM_001032905

R: AAAAGGAGTCAGATGTTTCGAGGT

IL-1β F: GACGTCGATGGCCCTAAACA NM_001042756 R: TGTAGTGCTCGTGGGAGATTTG

IL-4 F: ACAACTGCCATATCGCCTTACG NM_001032904 R: CTTCTGCAGGGCTGCGAC

IL-6 F: TGACAAACACATTCGGTACATCCT NM_001042733

R: AGCAAAGAGGCACTGGCAGA

IL-10 F: CCGTGGAGCAGGTGAAGAAT NM_001044727

R: GACATCTTCATCAACTACATAGAAGCCTA

CXCL11 F: AGAAAGCCTCCATAATTTACCCAAGT NM_001032950

R: GATTTGGGATTTAGGCATCGTT

LTA F: AGGATGGTTTCTCCTTGAGCAA NM_001047148

R: GGAGAGTAGGCTTTCCCAGAGAAG

CCL2 F: AGTGTCCCAAAGAAGCTGTGATC NM_001032821

R: TCCAGGTGGTCCATGGAATC

TNF-α F: TGAGGCCAAGCCCTGGTA NM_001047149

R: CGAGATAGTCGGGCAGATTGA

Arg-1 F: AACAGCTGGCTGGCAAGGT NW_001116523

R: TGGCCAGAGATGCTTCCAAT

CRP F: CTCATGCTTTTGGCCAGACA NW_001108960

R: GGCTTCGTTAACCGTGCTTT

Definition of abbreviations: F, forward primer; R, reverse primer.
