# Supplementary material for: Maternal High Fat Diet Is Associated with Decreased Plasma n–3 Fatty Acids and Fetal Hepatic Apoptosis in Nonhuman Primates
Source: PLoS One. 2011 Feb 25;6(2):e17261. doi: 10.1371/journal.pone.0017261 (PMC3045408; doi:10.1371/journal.pone.0017261)
Supplement: Methods S1 — Additional methods used in this study. (DOC) [file pone.0017261.s003.doc]

**METHODS S1**:

**Methods S1a.** Maternal insulin sensitivity. Twice a year, i.v. glucose tolerance tests (IVGTT’s) were performed on pregnant (early third trimester) and nonpregnant females after an overnight fast. Animals were sedated with ketamine (10 mg/kg) and administered a glucose bolus (50% dextrose solution) at a dose of 0.6 g/kg via the saphenous vein. Baseline blood samples were obtained prior to the infusion, and 1-ml blood samples were taken at 1, 3, 5, 10, 20, 40, and 60 min after infusion via the femoral artery. Glucose was measured immediately at each time-point using a OneTouch Ultra Blood Glucose Monitor (LifeScan), and the remainder of the blood was kept in heparinized tubes on ice for insulin measurement. After the IVGTT, samples were centrifuged, and plasma was stored at –80°C until assayed. Insulin was assayed in plasma by RIA (catalog no. RI-13K; Linco).

**Methods S1b.** Breast milk insulin, leptin, IL-1β and protein analysis. Insulin and leptin levels in the aqueous milk layer were assayed by commercially available primate radioimmmunoassay (RIA) kits (Insulin, Cat. # HI-14HK. leptin, Cat. # PL-84K; Linco, St. Charles, MO) according to the manufacturer’s instructions. Briefly, samples (100 µl) were assayed in duplicate and added to tubes with 100 µl of hydrated ^125^I-labeled human insulin or leptin and appropriate primate antiserum and incubated overnight at 4°C. Precipitating reagent (1 ml) was added, and tubes were centrifuged for 20 min at 2000-3000 *g*, then aspirated and total counts measured by gamma counter for 1 minute. The sensitivity of the assay was 2µU/ml for insulin and 0.5 ng/ml for leptin when using 100 µl of sample. IL-1β levels were determined using a monkey IL-1β ELISA (U-Cytech, Utrecht, The Netherlands) following the manufacturer’s specifications and assaying the samples in duplicate. Breast milk total protein levels were measured from the aqueous layer using BCA^TM^ Protein Assay Kit according to manufacturers specifications.

**Methods S1c.** RNA extraction protocol from liver tissue for Real-Time PCR. Total RNA was extracted from RNAlater (Ambion) stabilized primate fetal livers using an RNeasy Mini kit according to manufacturer’s instructions (Qiagen, Appendix C, June 2001) with the following additions: Following disruption and homogenization of liver tissue, a 15 min Proteinase K incubation at 55 ^o^C was performed. In addition, an optional on-column DNase treatment was also included. The RNA was eluted in RNase free water and the concentration and relative purity was determined by the 260/280 absorbance ratio. 1.3 ug of total RNA was reverse transcribed in a 75ul reaction using a Taqman Reverse Transcription kit (Applied Biosystems N808-0234) according to manufacturer’s protocol.

**Methods S1d.**  Real-Time PCR primer design. Target sequence was determined by using both the NCBI macaque database and the Ensembl macaque database. Sequences from each database were compared and only regions that had 100% homology between databases were used for primer design. Primers sets were designed by Primer Express (Applied Biosystems) to span exon-exon junctions where feasible and produce amplicons of ~100 bp in length.
